# Primary Thyroid Disorders in Patients with Endogenous Hypercortisolism: An Observational Study

**DOI:** 10.1155/2014/732736

**Published:** 2014-05-05

**Authors:** Eda Demir Onal, Muhammed Sacikara, Fatma Saglam, Reyhan Ersoy, Bekir Cakir

**Affiliations:** Department of Endocrinology and Metabolism, Yildirim Beyazit University Medical School Ataturk Teaching and Research Hospital, Bilkent, Sokak No. 10/6, Ceyhun Atıf Kansu Caddesi 1268, Ehlibeyt Mahallesi, 06520 Balgat, Cankaya, Ankara, Turkey

## Abstract

Cushing's syndrome (CS) may alter the performance of the hypothalamic-hypophyseal-thyroid axis. We searched for a relationship between hypercortisolism and primary thyroid disorders. The medical records of 40 patients with CS were retrospectively examined. Thyroid ultrasonography (USG), basal thyroid function test results (TFT), and antithyroglobulin and antithyroperoxidase antibodies were analyzed. In 80 control subjects, matched by age and gender with CS patients, thyroid USG, TFTs, and autoantibody panel were obtained. Among the CS patients, 17 had nodular goiter, versus 24 controls (42.5% versus 30%, *P* > 0.05). Among the twenty-five patients with an available TFT and autoantibody panel—before and after surgical curative treatment—autoantibody positivity was detected in 2 (8%) patients before and 3 (12%) after surgery (*P* = 0.48). Regarding TFT results, 1 (2.5%) patient had subclinical hyperthyroidism and 1 (2.5%) had subclinical hypothyroidism, whereas 1 (2.5%) control had hyperthyroidism. In total, 21 (52.5%) patients and 32 (40%) controls had ≥1 of the features of thyroid disorder, including goiter, positive thyroid autoantibody, and thyroid function abnormality; the difference was not significant (*P* > 0.05). The prevalence of primary thyroid disorders is not significantly increased in patients with CS.

## 1. Introduction

Endogenous glucocorticoids (GCs) have diverse anti-inflammatory and immune modulatory actions. These effects are mediated through a number of distinct mechanisms which include actions on leukocyte populations, interfering with their trafficking, chemotaxis, phagocytosis, and inflammatory activation. They also reduce the synthesis and release of many secreted mediators of inflammation [1]. Endogenous Cushing's syndrome (CS) is a nonphysiological hypercortisolism state which causes a reversible state of immunosuppression [2]. Autoimmune diseases have improved during the active phase of CS whereas there is a risk of worsening of the same conditions upon remission [3–5]. CS may alter the performance of the hypothalamic-hypophyseal-thyroid axis in several ways as well [6]. In this study we aimed to determine if there is a relationship between hypercortisolism and primary thyroid disorders, focusing mainly on autoimmune thyroiditis.

## 2. Materials and Methods

The medical records of 52 CS patients admitted to our endocrine unit from 2006 to 2013 were retrospectively examined. CS cases with a presurgical record of thyroid ultrasonography (USG) and at least one measurement of basal serum thyroid stimulating hormone (TSH), circulating free thyroxine (fT4), free triiodothyronine (fT3), antithyroglobulin (anti-Tg), and/or antithyroperoxidase (anti-TPO) antibodies were selected for inclusion. Forty patients met these minimum requirements. When available, data on thyroid function tests (TFTs) and autoantibody panel 6 months after surgery were also evaluated. Diagnosis of hypercortisolism had been established by an overnight low dose dexamethasone suppression test and urinary free cortisol (UFC) was measured in 24-h samples [7–9]. Etiological diagnosis was made by 8 mg dexamethasone suppression test, measurement of ACTH levels, and imaging techniques (sellar region magnetic resonance imaging and/or lung and adrenal computed tomography scan) [10]. Inferior Petrosal sinus sampling with corticotropin releasing hormone stimulation was performed in 13 patients to confirm pituitary origin. Cure of CS was established on the basis of the following criteria: (1) urinary daily cortisol excretion and plasma ACTH concentrations below or within the normal range, (2) serum cortisol concentrations below or within the normal range with restoration of physiological circadian rhythm, and (3) suppression of urinary and serum cortisol concentrations after low dose dexamethasone test [11].

Serum fT4, fT3, TSH, anti-Tg, and anti-TPO levels had been measured by immunoassay using commercial kits. The normal ranges were fT3: 1.8–4.6 pg/mL, fT4: 0.9–1.7 ng/dL, TSH: 0.27–4.2 uIU/mL, anti-Tg: 0–115 IU/mL, and anti-TPO: 0–115 IU/mL. Anti-Tg and anti-TPO titres 3 standard deviations above the mean were considered as positive. The thyroid USG was performed on a General Electric RT3600 scanner using a 7.5 MHz linear array transducer and a direct contact technique by an experienced endocrinologist. Goiter was diagnosed when the anteroposterior diameter in both lobes was ≥20 mm. The presence of a diffuse hypoechoic pattern with or without hyperechoic lines was considered suggestive of thyroiditis.

A control group, matched by age and gender with CS patients, was used for this study and procedures were applied in agreement with the local ethics committee. All subjects denied having known thyroid diseases or relatives with any kind of thyroid disorders. In the control group, TFTs and autoantibody panel were obtained and thyroid USG was performed.

Statistical analysis was performed by SPSS software (Statistical Package for the Social Sciences, version 18.0, SPSS Inc., Chicago, IL, USA). Data were expressed as means ± standard deviation. Comparisons between categorical data were preformed by Chi square test with Yates' correction. With respect to numerical data, comparisons between the patients and controls were performed by Student's *t*-test. Paired-samples *t*-test was used to compare numerical data before and after surgery. The *P* values were given for these analyses. Significance was set at 5%.

## 3. Results

The study included a patient group (*n* = 40) and a control group (*n* = 80). There were no significant differences between the patient and control groups with respect to sex and age (male/female ratio 9/31 versus 16/64, mean age 49.9 ± 12.7 versus 46.4 ± 8.7 years, resp., *P* > 0.05). The etiology of CS was pituitary in 18 patients, adrenal in 21, and bronchial carcinoid in 1. All 21 patients with CS due to adrenal adenoma were successfully treated with unilateral adrenalectomy and the 1 patient with ectopic ACTH syndrome was cured following resection of a bronchial carcinoid. All 18 patients with CS due to pituitary adenoma underwent surgical selective resection of the tumor via the transsphenoidal approach: histology and immunohistochemistry results of the surgically removed pituitary adenomas confirmed the diagnosis of CS in all cases. After surgery, 16 patients had remission of disease, whereas the remaining 2 had persistent disease and underwent unilateral adrenalectomy due to coexistent adrenal adenoma. Regarding all CS patitents, the mean urinary cortisol excretion was 357.59 ± 98.76 mcg/24 h at the initial diagnosis and 48.42 ± 26.75 mcg/24 h after curative treatment.

TFT results were compatible with euthyroidism in 38 (95%) patients with active CS. There was 1 (2.5%) patient with subclinical hyperthyroidism and 1 (2.5%) with subclinical hypothyroidism. Following cure of CS, TFT results were available in 32 patients, all of which were euthyroid, except for 3 (9%) patients with subclinical hyperthyroidism. All the controls were euthyroid, except for 1 (2.5%) with toxic nodular goiter. There was no significant difference between the three groups with respect to TFTs (*P* > 0.05). Thyroid USG showed nodular goiter in 17 (42.5%) patients with active CS and in 24 (30%) controls (*P* > 0.05). Thyroid USG was indicative of a pattern of thyroiditis in 7 (17.5%) patients with active CS and in 28 (35%) controls (*P* > 0.05). Thyroid autoantibody panel findings are shown in Table 1. Among the twenty-five patients with an available TFT and autoantibody panel—before and after surgical curative treatment—autoantibody positivity was detected in 2 (8%) patients before and 3 (12%) after surgery (*P* = 0.48). Thyroid autoantibodies were positive in 8 out of 40 patients, 2 of which had new onset autoantibody positivity after cure of CS. The characteristics of 8 CS patients with evidence of thyroid autoimmunity during followup are shown in Table 2. In total, 21 (52.5%) patients and 32 (40%) controls had ≥1 of the features of thyroid disorder, including goiter, positive thyroid autoantibody, and thyroid function abnormality; the difference was not significant (*P* > 0.05). Subgroup analysis of the patients with nodular goiter—based on the etiology of CS—showed that 12 (52.2%) of 23 patients with an adrenal tumor and 4 (22.2%) of 18 patients with CS had nodular goiter. There was a significant difference in the presence of goiter between patients with an adrenal tumor and the controls (*P* < 0.01).

## 4. Discussion

Increasing evidence indicates that the immune and endocrine systems are linked. Cytokines produced by activated immune and immune accessory cells can affect—positively or negatively—the secretion of hormones from the hypothalamic-pituitary-adrenal axis. The main pituitary-dependent hormones that affect immune reactions are GCs. GCs suppress inflammatory response genes and regulate cytokine genes. Persistent hypercortisolism induces lymphopenia and lymphoid tissue atrophy [12]. Correction of hypercortisolism can be followed by a rapid increase in immune function, which can exacerbate a preexisting autoimmune disease or precipitate an autoimmune disease in predisposed individuals [13]. But, interestingly, few studies have evaluated the effect of resolution of hypercortisolism on immune function.

Colao et al. retrospectively analyzed the occurrence of autoimmune thyroid diseases in 20 patients with CS after successful surgical treatment [14]. They reported an increase in the prevalence of anti-thyroid autoantibodies of up to 60% at the sixth month following cure, as compared to 20% before cure. They reported that 25% of their patients developed hypothyroidism—primarily subclinical—always in association with anti-thyroid autoantibodies following Cushing's treatment. In 40% of patients there was new onset of serum anti-thyroid autoantibodies. In their series of patients with endogenous CS, Niepomniszcze et al. observed a significantly higher frequency of goiter (30.5 versus 8.5%), thyroid autoimmunity (56.1 versus 10%), and subclinical hypothyroidism (23.7 versus 2.5%) in the patients than in the control group [15]. The overall frequency of primary thyroid abnormalities in their patients with CS was 55.9%. In that study, followup (mean: 9.8 months; range: 2–18 months) showed that the prevalence of positive anti-thyroid antibodies during active disease (26.7%) increased sharply (86.7%) after hypercortisolism was resolved. Lastly, Invitti et al. observed a significantly higher prevalence of thyroid nodular disease in patients with CS than in controls (60.0% versus 20.0%) [16].

Anti-TG and anti-TPO are associated with thyroid inflammation, and detection of these autoantibodies is a very specific means of diagnosing autoimmune thyroid disease [17, 18]. Thyroid autoantibodies were reported to be positive in 18–31% of the healthy population according to previous studies from Turkey which is a mild-to-moderate iodine deficient area [19–24]. In the present study thyroid autoantibodies were detected in 15% of the controls. There was not a difference in the frequency of anti-thyroid autoantibody positivity between the patients with endogenous hypercortisolism and the controls (15% versus 15%, *P* > 0.05). In addition, only 2 patients had new onset autoimmune thyroid disease, whereas autoantibodies were negative in 1 patient after CS was cured. Our results do not agree with those of the above-mentioned studies which suggest an association between autoimmune thyroid disease and CS [14, 15]. There may be several explanations for this conflict. Ideally, a study investigating such an association should be a prospective randomized controlled trial with a large number of participants, while also taking into consideration several possible confounding factors such as dietary iodine intake, personal or family history of autoimmune disease, certain medications (e.g., immunosuppressants, amiodarone), and radiation exposure. None of the above-mentioned studies [14, 15] provide irrefutable evidence in support of a direct causative link between autoimmune thyroid disease and CS. The amazing complexity of the immune system may also argue against such a simple relationship between these two entities. For example the functional activity of T cell lymphocytes remains normal at least in ACTH-dependent Cushing's patients compared with controls in spite of the inhibitory effect of glucocorticoids on T cell lymphocyte proliferation [25]. And to our knowledge there is not any definitive laboratory evidence or animal studies which prove a tendency to develop autoimmune thyroid disease during resolution of CS.

The frequency of nodular goiter was previously reported to be between 5 and 56% in Turkey [19, 21, 23, 26]. It was 30% in the present study and there was no significant difference between the CS patients and control subjects with respect to the frequency of nodular goiter (42.5% versus 30%, *P* > 0.05), which is not in agreement with earlier findings [14–16]. It has been proposed that CD cases may present an increased proliferation of thyrocytes through raised interleukin-6 levels secreted by tumoral corticotropic cells [27, 28]. Invitti et al. reported an increased prevalence of nodular goiter in CD patients. But with respect to adrenal tumors the prevalence was only slightly higher than that observed in control subjects [16]. The authors postulated that a growth factor stimulating both corticotroph and thyrocyte proliferation might be involved. On the other hand the number of patients with adrenal tumor was only 6 in this series. A higher frequency of goiter in patients with adrenal tumor in our series does not support this costimulatory-signal hypothesis. Endocrinologists are more inclined to screen patients with CS under their care for thyroid dysfunction and thyroid nodules than general practitioners, and we think this may explain to some extent the higher prevalence of primary thyroid disorders in this particular group of patients. In the present study, the decrease in serum-free triiodothyronine levels might have been associated with inhibition of T4 monodeiodination in peripheral tissues due to increased action of GCs.

There are several limitations in our study. First, our study has a retrospective design. Second, postoperative reevaluation of hormonal and antibody profile could be performed in 62.5% of patients six months after having cured their CS. Although this seems to be a reasonable time to expect changes, it is less than the time of monitoring carried out in the two main previous studies cited [14, 15]. Thus, the study does not have such a design to permit for data on the possible late development of autoimmune thyroid disease. Additionally, thyroid USG would have provided complementary information if it had been repeated as a part of the postoperative reevaluation. Third, thyroid autoantibody titers were not quantified in our series. Finally, we have a high proportion of patients with CS caused by an adrenal adenoma which is not consistent with the literature [15]. This may be because the patients with adrenal adenoma are more frequently referred to our hospital than the patients with pituitary tumor due to local expertise in pituitary and adrenal surgery. Despite the limitations, literature on the primary thyroid disorders in patients with CS is still scarce and we believe that our study provides additional evidence on this topic because it includes a relatively large number of CS patients with a high proportion of adrenal adenoma.

In conclusion, the present findings showed that primary thyroid disorders, including thyroid autoimmunity, did not occur more frequently than in the controls. Autoimmunity is a complex process and the results of similar studies should be cautiously interpreted. Future prospective studies with larger populations are needed to further clarify if there is a causative link between CS and primary thyroid disorders, especially autoimmune disorders.

## Figures and Tables

**Table 1:** Thyroid function tests and autoantibody panel of the patients with Cushing's syndrome and control subjects. Group 1A corresponds to the patients before cure whereas Group 1B includes the patients after cure for Cushing's syndrome. Group 2 is the control group.

	Group 1A (*n* = 40)	Group 1B (*n* = 25)	Group 2 (*n* = 40)	(*P*) Group 1A versus 2	(*P*) Group 1B versus 2	(*P*) Group 1A versus 1B
Serum TSH	1.3 ± 1.2	1.6 ± 1.2	2.2 ± 1.3	<0.01	0.06	0.223
Serum fT3	3.1 ± 0.8	3 ± 0.8	3.4 ± 0.7	0.09	0.035	0.814
Serum fT4	1.2 ± 0.3	1.2 ± 0.4	1.3 ± 0.2	0.4	0.442	0.646
Anti-Tg positivity (%)	4 (10)	4 (16)	3 (7.5)	0.692	0.253	0.371
Anti-TPO positivity (%)	4 (10)	5 (20)	5 (12.5)	0.723	0.322	0.221
Antibody positivity (%)^a^	6 (15)	7 (28)	7 (17.5)	0.762	0.244	0.169

TSH: thyroid stimulating hormone; fT3: free triiodothyronine; fT4: free thyroxine; anti-Tg: antithyroglobulin antibody; anti-TPO: antithyroperoxidase antibody. Statistical significance was set at a *P* value of 5%. Numerical data was expressed as mean ± SD. Normal values: fT3: 1.8–4.6 pg/mL, fT4: 0.9–1.7 ng/dL, and TSH: 0.27–4.2 uIU/mL. ^a^Positivity of anti-Tg and/or anti-TPO.

Data on thyroid function tests (TFTs) and autoantibody panel were evaluated 6 months after curative surgery.

**Table 2:** Characteristics of 8 patients with Cushing's syndrome who had evidence of thyroid autoimmunity during followup.

Patient (sex/age)	Pretreatment antibody profile		TFTs	Thyroid USG	Posttreatment antibody profile		TFTs	Urinary cortisol levels (mcg/24 h)	
	Anti-Tg	Anti-TPO			Anti-Tg	Anti-TPO		Before cure	After cure
1 (f/62)	Pos	Pos	Euthyroid	Normal	Pos	Pos	Euthyroid	180	44
2 (f/58)	Pos	Neg	Subclinical hypothyroid	Nodular goiter	Pos	Neg	Euthyroid	152	41
3 (f/33)	Neg	Pos	Euthyroid	Thyroiditis	Neg	Pos	Euthyroid	259	45
4 (f/52)	Neg	Pos	Subclinical hyperthyroid	Thyroiditis	Neg	Neg	Euthyroid	151	57
5 (m/63)	Pos	Pos	Euthyroid	Thyroiditis	Pos	Pos	Euthyroid	130	74
6 (f/45)	Neg	Pos	Euthyroid	Nodular goiter	Neg	Pos	Euthyroid	144	65
7 (f/26)	Neg	Neg	Euthyroid	Nodular goiter	Pos	Neg	Euthyroid	145	53
8 (f/58)	Neg	Neg	Euthyroid	Thyroiditis	Neg	Pos	Euthyroid	210	65

TFTs: thyroid function tests; f: female; m: male; anti-Tg: antithyroglobulin antibody; anti-TPO: antithyroperoxidase antibody; pos: positive; neg: negative.

Data on thyroid function tests (TFTs) and autoantibody panel were evaluated 6 months after curative surgery.
